# Post-Mortem Meat-Inspection of Swine

**Published:** 1900-02

**Authors:** W. E. Howe

**Affiliations:** Assistant Meat-Inspector, Chicago, Ill.


### POST-MORTEM MEAT-INSPECTION OF SWINE.

By W. E. Howe, V.S., D.V.M.,
ASSISTANT MEAT-INSPECTOR, CHICAGO, ILL.

In dealing with this subject of meat-inspection perhaps it will be well, for the benefit of those present who have not witnessed the killing of hogs on a large scale, to give a little idea of how the work is accomplished.

The inspector is on the bench with the workmen who remove the viscera from the carcass. The hogs are hung on an iron rail by means of a pulley, which has a double hook to catch the gambol-stick. They are then moved along the rail by means of an endless chain running parallel to the rail, which has fingers projecting to catch behind the pulley and move it along, so the hog is in constant motion. The inspector is provided with a seat, usually at the end of the bench and so situated that the hogs pass on review so closely that the inspector can touch them without rising from his seat. The viscera are dropped on the bench beside him, where he has a good view. Thus the hogs pass in different houses at a rate of from two hundred to seven hundred an hour. The inspector keeps watch, and any carcass that shows evidence of disease is tagged. The viscera are also tagged and saved, the two tags bearing duplicate numbers, so the viscera can be readily found that belong to any hog in question. The diseased carcasses are run into a separate place from the others, so that they can be examined closely after the day's work is finished. Then they are disposed of as the case demands, either put into the offal-tank or released.

In this paper time will not permit me to cover all the diseased conditions with which we come in contact, so will only touch upon a few. But, first, what good reasons have we for condemning any of these diseased conditions which we find?

1. For sentimental reasons.

2. There is a sufficient amount of good meat in this country that can be obtained at reasonable figures, so we do not need to eat any but what is free from disease.

3. For the effects it would produce upon the one consuming the meat.

As many of the diseases with which we come in contact most frequently are of bacterial origin, it brings up the question of "How do bacteria produce disease?" Many theories have been brought forward at different times, but the theory which now is generally accepted is that symptoms and disease are caused by the chemic poisons produced by the microörganisms. Here three possibilities present themselves: First, the organisms themselves may be poisonous, or the poison may be an integral part of them. Second, the microörganisms may be intimately associated with or may produce a soluble chemic ferment which by its action on the body produces the symptoms of the disease and death. Third, poisons may be produced by the cellular activity of the bacteria. The correctness of this theory has been tested by a large number of investigators with the result that its correctness has been fully established. It is now known that the pathogenic germs grown in meat-broth and other culture media elaborate chemic poisons which when injected into animals induce in an acute form one or more of the symptoms of the disease caused by the microörganism. It can now be safely stated that every germ which produces disease does so by virtue of its chemic poisons.

The diseases most frequently found by the inspector are hog-cholera, swine-plague, tuberculosis, metritis, peritonitis, pleurisy, pneumonia, bruises, tumors, nephritis, and hogs that have died just previous to the time of slaughter.

We will turn our attention for a few minutes to hog-cholera and swine-plague, which are by far the most frequent causes for condemnation. The causes of these diseases are bacilli differing in many ways and alike in many respects: the bacillus cholera suis and the bacillus of swine-plague, which I will not describe.

In giving the pathological lesions those only that may be readily seen by the inspector in his hurried examination will be dwelt upon.

Probably the first noticed are the red spots which appear upon the surface. They may be small spots a little larger than a pin-head, but close together, or much larger ones the size of a pea; others may be close together, perhaps coalesced to form still larger ones, and I have seen other severe cases with scarcely any spots at all upon them. Still other cases will have a diffuse redness around the scrotal region and a red line along the under side of the abdomen. In some it will be confined principally to the ears, which will be much swollen and covered with red spots. In the majority of cases the spots will be thicker and better defined on the inner and softer portions of the skin.

The appearance of the intestines varies much with the severity and stage of the disease at the time it is seen. As they are dropped upon the bench beside the inspector they may not attract any particular attention. The lymphatic glands of the mesentery may be inflamed. If the intestine is slit the punctiform hemorrhages may be found, or in more severe cases the button-like ulcers; still others will show the mucosa sloughed away for a long distance and the inflammation extending through the wall of the intestine, showing severe inflammation and extravasation of blood into the mesentery and the surrounding fat. The kidneys also have small punctiform hemorrhages in the cortex and frequently in the extremities of the pyramids. The peritoneum occasionally presents hemorrhagic areas, especially upon the sides, and easily noticed against the fat which forms a white background. The spleen is enlarged, swollen, full of blood, very soft and brittle, dark-colored, almost black. The lungs present a varying appearance, sometimes only a few small areas of congestion will be seen, while in others larger portions will be involved in a pneumonia. Punctiform hemorrhage may also be seen on the base of the tongue, on the heart, and in severe cases in nearly all parts. The lymphatic glands throughout the system are much enlarged, swollen, congested, and when cut appear to be full of blood. Sometimes the glands will appear to be inflamed only upon the surface. The bones appear normal upon the surface, but when cut the cancellated structures will appear very black.

In swine-plague the lesions are so similar to those of hog-cholera that it is hard to distinguish one from the other, and the two diseases are seen together in the same animal so frequently that it cannot be done in the short space of time allowed us for examination. The principal difference seems to be that in swine-plague the disease begins in the lungs and spreads from there to other parts, while in hog-cholera it appears to have started in the intestines and extended from there to other parts. But the pathological lesions are so similar that a positive post-mortem diagnosis cannot be made without the aid of the microscope.

Why do we condemn for this disease? First, for sentimental reasons ; second, we find that the hog-cholera bacillus produces a ptomaïn which has been called sucholotorin, and a proteid substance called suchaloalbumin. Experimentally these substances in very small doses are not fatal to experimental animals, but in larger doses they will produce death with some of the prominent symptoms of hog-cholera. A properly regulated dose will produce a sickness for a short time, when recovery will take place, and afterward the animal will show some immunity from the disease.

Dead hogs. One difficult point ofttimes is to be able to say absolutely whether a hog has died a short time before it was stuck and scalded, or whether it was poorly stuck, so it did not bleed out well, and consequently fell into the tub alive and was drowned while being scalded. In both cases when the animal reaches the inspector it will show that it has not bled out well. The lungs, kidneys, and liver will be full of blood in both cases. If the hog was poorly stuck and dropped into the scalding tub alive, there would still be the peristaltic action of the bowels, some twitching of the muscles, and possibly some beating of the heart, while in a dead hog these would be absent. Sometimes in cases it is difficult to make a clear distinction. If they are permitted to hang in the cooler for a few days, the dead one will show signs of decomposition. The dead ones are always condemned, no matter how well they may appear after they are dressed and hung in the cooler. The hogs frequently die from exhaustion, heat, and asphyxiation. In these cases we would have in the flesh a large amount of the waste matters of the body which have not been thrown off, and being full of blood putrefaction soon starts, consequently the flesh would be filled with the ptomaïns of putrefaction which are putrescine and cadaverin. These ptomaïns are capable of producing strong inflammation and necrosis. Cadaverin is one of the substances which can set up suppuration in the absence of bacteria. In Asiatic cholera the necrosis of the intestinal epithelium and the muscular spasm are thought to be due to this same product.

In tuberculosis the pathological lesions of the diseases are so well known that I will only make a few remarks, which will apply especially to the disease as found in swine. This disease in swine originates most commonly from indigestion, so that the disease does not appear first and most prominently in the lungs and respiratory tract, but in the digestive organs and their accessory glands, principally in the liver and spleen. Here it is seen either as miliary granulations scattered in great numbers throughout the thickness of the organ, or else of rounded nodosities, which are yellowish-white in color, varying in size from that of a pea to that of a hazelnut. On section they appear sometimes softened in the centre, but rarely infiltrated with calcareous salts. Lesions similar to those of the liver may be seen in the lungs in severe cases, but generally there is found in these organs an innumerable quantity of minute gray granulations caused by generalization through the blood-stream, in which case the liver, the spleen, the kidneys, the medulla of bones, and the mammæ are usually infiltrated with similar growths. Cases are sometimes seen in which the disease appears to be localized in a few glands. The tonsils and the pharyngeal or submaxillary glands are the ones most often affected. They become voluminous, hard, and knotty, as they have undergone a true fibrous transformation ; here and there small yellow foci are seen of a soft consistency, sometimes veritable purulent collections are found, either encysted or in communication with the interior. If the caseous or purulent matter be subjected to any examination tubercle bacilli are not usually found, but upon inoculation they are found to be present. The generalization of the disease, especially in the muscular tissue, is reported by several observers.

Why do we condemn for this disease ? I think there is not one present at this meeting who would hesitate to condemn a hog for tuberculosis. I condemn every one found that shows symptoms of the disease, because in swine it becomes generalized so quickly, and the result of experiments upon this subject show that muscle-juice of tuberculous swine is often virulent. Another point worthy of consideration is the increased danger from the consumption of meat from tuberculous swine over tuberculous cattle, which is due to its greater tendency to become generalized in swine. The consumption of tuberculous pork by a person inclined to be tuberculous or who is afflicted with the disease in a latent stage would be like taking a small dose of tuberculin, and it produces the same effect —that is, to awaken the disease afresh and start it on in its deadly course.

BIBLIOGRAPHY.

The United States Bureau of Animal Industry Reports.
Vaughan and Novy : "Ptomaïns, Leucomaïns, Toxins, and Antitoxins."

